# Population pharmacokinetic analysis of TQ-B3203 following intravenous administration of TQ-B3203 liposome injection in Chinese patients with advanced solid tumors

**DOI:** 10.3389/fphar.2023.1102244

**Published:** 2023-01-16

**Authors:** Xiaoqing Li, Yunhai Bo, Han Yin, Xiaohong Liu, Xu Li, Fen Yang

**Affiliations:** ^1^ Key laboratory of Carcinogenesis and Translational Research (Ministry of Education), National drug clinical trial center, Peking University Cancer Hospital & Institute, Beijing, China; ^2^ Chia Tai Tianqing Pharmaceutical Group Co Ltd, Nanjing, Jiangsu, China

**Keywords:** population pharmacokinetic model, camptothecin, TQ-B3203, model validation, model application

## Abstract

**Background:** TQ-B3203 is a novel topoisomerase I inhibitor currently in development for the treatment of advanced solid tumors. Great differences in pharmacokinetic characteristics were found among individuals according to the phase I clinical trial following intravenous administration of TQ-B3203 liposome injection (TLI) in Chinese patients with advanced solid tumors. Thus, it is significant to establish a population pharmacokinetic model to find the key factors and recognize their effect on pharmacokinetic parameters in order to guide individualized administration.

**Methods:** Non-linear mixed effect models were developed using the plasma concentrations obtained from the phase I clinical trial by implementing the Phoenix NLME program. Covariates that may be related to pharmacokinetics were screened using stepwise methods. The final model was validated by goodness-of-fit plots, visual predictive check, non-parametric bootstrap and a test of normalized prediction distribution errors.

**Results:** A three-compartment model with first-order elimination was selected as the best structural model to describe TQ-B3203 disposition adequately. Direct bilirubin (DBIL) and body mass index (BMI) were the two most influential factors on clearance, while lean body weight (LBW) was considered to affect the apparent distribution volume of the central compartment. The population estimations of clearance and central volume were typical at 3.97 L/h and 4.81 L, respectively. Model-based simulations indicated that LBW had a great impact on C_max_, BMI exerted a considerable influence on AUC_0-t_, and the significance of DBIL on both AUC_0-t_ and C_max_ was similarly excellent.

**Conclusion:** The first robust population pharmacokinetic model of TQ-B3203 was successfully generated following intravenous administration of TLI in Chinese patients with advanced solid tumors. BMI, LBW and DBIL were significant covariates that affected the pharmacokinetics of TQ-B3203. This model could provide references for the dose regimen in the future study of TLI.

## 1 Introduction

Topoisomerase I, a critical intra-nuclear enzyme for DNA replication, has a higher activity and replication rate in most cancer cells. Camptothecin (CPT), extracted from traditional Chinese medicine prescriptions (Redinbo et al., 1998), is a topoisomerase I inhibitor that could combine topoisomerase I and DNA complex to form a stable ternary complex to prevent DNA reconnection, cause DNA damage, introduce G2/M phase arrest, and therefore lead to cancerous cell apoptosis (Hertzberg et al., 1989; Redinbo et al., 1998). CPT and its derivatives have played an essential role in the treatment of some solid tumors, including colorectal cancer, liver cancer, small-cell lung cancer and glioblastoma (Pommier, 2006; Selas et al., 2021).

Irinotecan, the most representative chemically modified CPT derivative, was created to avoid the poor water-solubility, low antineoplastic activity (Gottlieb and Luce, 1972) and many adverse reactions of CPT (Rozencweig et al., 1976). Although it has been clinically used in anticancer treatments for nearly 28 years after approval by the FDA, some obvious disadvantages still limit its application, such as short half-life and high toxicity (Herben et al., 1996; Chabot, 1997; Pommier, 2006). TQ-B3203 is a novel semisynthetic derivative of CPT with an aliphatic chain. It exhibited more vital pharmacodynamic activity than irinotecan in the tumor growth inhibition test of several cell types (Zhang et al., 2017) because it could accumulate more in cells to increase cytotoxicity due to its higher lipophilicity. Furthermore, TQ-B3203 liposome injection (TLI) was produced after TQ-B3203 was embedded in liposomes in order to acquire a significant reduction in toxicity and improvement of efficacy because liposomes, well-recognized drug delivery carriers, have the ability to prolong drug circulation time, passively accumulate in tumor tissues and increase drug exposure (Drummond et al., 1999; Yang et al., 2013). The formulation-related stability and the distribution *in vivo* were evaluated in the pre-clinical study (Supplementary Figure S1, S2 in Supplementary Material).

The narrow therapeutic window is a critical defect of cytotoxic anticancer drugs, thus their toxicity has a strong correlation with drug exposure, which depends on the pharmacokinetic characteristics. Therefore, it is necessary to find key factors leading to pharmacokinetic variability among patients (Ardizzoni et al., 1997). Population pharmacokinetic (PopPK) modeling is a standard method to identify the essential determinants of drug disposition by using the drug concentration and covariate information from subjects, such as demographics, clinical laboratory results and genetic characteristics. In previous PopPK studies of irinotecan, some covariates are considered to have significant effects on pharmacokinetic parameters, such as performance status and liver function on clearance (CL) and body weight on apparent distribution volume of the central compartment (V_1_) (Klein et al., 2002; Mathijssen et al., 2002; Xie et al., 2002). As an analog of irinotecan, TQ-B3203 also showed great differences in pharmacokinetic characteristics among individuals according to the phase I clinical trial, so it is significant to find the potential covariates on pharmacokinetic parameters in order to guide individualized administration.

In this study, we aimed to develop a PopPK model of TQ-B3203. The influence of selected internal and external factors on pharmacokinetics was recognized and quantified to characterize the pharmacokinetic difference among individuals. Additionally, the effects of significant covariates on TQ-B3203 exposure in patients were also explored. This study will provide critical information for the future development and clinical application of TQ-B3203.

## 2 Methods

### 2.1 Clinical trial

This multi-center, dose-escalation phase I clinical study (Register No. NCT03447145) was conducted to assess the pharmacokinetic characteristics and safety of TQ-B3203 after TLI intravenous administration. This study was designed in accordance with the ethical principles described in the Declaration of Helsinki and approved by the Ethics Committee of Peking University Cancer Hospital. All patients signed informed consent prior to their enrollment in this study. The process of this phase I clinical study did not involve randomization, blinding and power analysis, and the attrition was recorded.

Demographic characteristics were collected from electronic medical records. Patients aged 18–80 years with clearly diagnosed advanced solid tumors were enrolled in this study. Other key inclusion criteria were as follows: body mass index (BMI) of 18.5–26 kg/m^2^, Eastern Cooperative Oncology Group (ECOG) performance status of 0–1, life expectancy > 3 months, normal primary organ functions and >30 days recovery after receiving anti-tumor treatment or surgery.

Patients were not eligible if they have suffered from other malignant tumors within 5 years, have participated in other clinical trials within 4 weeks, have received other CPT analog therapy, and were in possession of neurological, circulatory and urinary diseases such as meningitis, pericardial effusion, coagulopathy or hypertension.

### 2.2 Drug administration and sampling

The novel anticancer drug TLI was provided by Nanjing Chia-tai Tianqing Pharmaceutical Group (Nanjing, China). Patients received TLI with dose levels ranging from 2 to 45 mg/m^2^ by intravenous infusion for 90 min on days 1 (cycle 1) and 22 (cycle 2). Blood samples were collected at pre-dose, 45 and 90 min after the infusion start, and were obtained at .5, 1, 2, 4, 8, 12, 24, 48, 72 and 96 h after the end of the infusion. The whole blood samples were immediately centrifuged at 3,000 rpm for 10 min at 4°C and obtained plasma samples were stored at −80°C pending analysis.

### 2.3 Analytical methods

The plasma concentration of TQ-B3203 in this study was determined using a fully validated liquid chromatography-tandem mass spectrometry (LC-MS/MS) method with bis (p-nitrophenyl) phosphate (2 mol/L) used as the esterase inhibitor and TQ-B3203-d_8_ used as the internal standard (the structures of TQ-B3203 and TQ-B3203-d_8_ are shown in Supplementary Figure S3 in Supplementary Material), which has been reported in the previous study (Yang et al., 2021). The plasma samples were protein precipitated by methanol and the processed samples were chromatographed on an AQUITY BEH C8 column (50 × 2.1 mm, id 1.7 μm) with acetonitrile and water (.1% formic acid) as the mobile phase. Mass spectrometric analysis was performed on Waters Xevo TQS tandem mass spectrometer (Waters Corp. Milford, MA, United States) equipped with an electrospray ionization source in positive mode (ESI+). The ESI source settings were as follows: Capillary voltage, 4.0 kV; Source temperature, 150°C; Desolvation temperature, 500°C; Cone gas flow, 150 L/h; Desolvation gas flow, 1000 L/h; Nebulizer gas pressure, 7 bar. Multiple reaction monitoring (MRM) transitions and related collision energy were m/z 949.5→393.1 (58 eV) for TQ-B3203 and m/z 957.4→398.0 (50 eV) for TQ-B3203-d_8_. The linear range of TQ-B3203 was .5–500 ng/mL. Accuracy and precision were within the acceptable range of FDA bioanalytical assay validation criteria (e.g., ± 15%).

### 2.4 Pharmacokinetic study and statistical analyses

The pharmacokinetic parameters such as CL, the apparent volume of distribution (V_z_), elimination half-life (t_1/2_), the area under the plasma concentration-time curve from zero to the last time (AUC_0-t_) and from zero to infinity (AUC_0-inf_) were analyzed and calculated by the non-compartmental analysis (NCA) using the Phoenix (RRID:SCR_003163) WinNonlin (version 8.3, Pharsight Corporation, CA, United States), except that maximum observed plasma concentration (C_max_) and time to C_max_ (T_max_) were obtained directly from the observed concentration. The pharmacokinetic parameters (CL, V_z_, t_1/2_ and AUC_0-t_) between cycles 1 and 2 were compared using paired t-tests, with significance denoted by *p* < .05.

### 2.5 PopPK modeling

The PopPK analysis of TQ-B3203 was performed using non-linear, mixed-effect modeling of Phoenix (RRID:SCR_003163) NLME (Version 8.3, Pharsight Corporation, CA, United States). R program (Version 4.2.0, R Project for Statistical Computing, RRID:SCR_001905, http://www.r-project.org/) was used for statistical summaries and graphical analysis. The first-order conditional estimation-extended least-squares (FOCE-ELS) method built into the modeling program was applied for the estimation of pharmacokinetic parameters, covariate testing and model diagnostic.

#### 2.5.1 Basic model

According to the semilogarithmic plots of individual TQ-B3203 plasma concentration-time, two- and three-compartment models with first-order elimination were tried to describe the dataset. Inter-individual variability (IIV, η, eta) was estimated by an exponential model, which was shown in Eq. 1:

$$P_{ij}=\theta_{i}*e^{\eta_{ij}}$$ (1)

where $P_{ij}$ represents the individual pharmacokinetic parameter estimation for *i*th parameter in *j*th individual, $\theta_{i}$ is the typical parameter estimation value for *i*th parameter and η_ij_ depicts the random variable for *i*th parameter in *j*th individual. Intra-individual variation, also known as residual variation (ε, epsilon), was tested by employing the additive, log-additive, proportional and power models. The distributions of η and ε were considered to follow a Gaussian distribution with mean of 0 and variance of ω^2^ (omega) or σ^2^ (sigma) as diagonal matrixes, respectively.

Inter-occasion variability (IOV) on the CL and the V_1_ between Day 1 in cycles 1 (occasion 1) and 2 (occasion 2) was estimated in the basic model before the covariate selection. It was also included in the model as an exponential term (η_IOV_) in Eq. 2 and Eq. 3 (Karlsson and Sheiner, 1993):

$$P_{ij}=\theta_{i}*{e^{\eta_{ij}}*e}^{\eta_{IOV,1}}$$ (2)

$$P_{ij}=\theta_{i}*{e^{\eta_{ij}}*e}^{\eta_{IOV,2}}$$ (3)

in which $\eta_{IOV,1}$ and $\eta_{IOV,2}$ are variabilities between occasions for the *i*th parameter in *j*th individual on occasion 1 and on occasion 2, respectively.

The model superiority was determined by better visual inspection of the diagnostic plots and a smaller diagnostic index such as objective function (OFV), Akaike Information Criteria (AIC) and Bayesian information criteria (BIC). It was considered statistically significant that the OFV value (1 degree of freedom in χ2 distribution) was decreased by ≥ 3.84 points (*p* < .05).

#### 2.5.2 Covariate analysis

Several covariates used for covariate analysis were directly obtained from the electronic medical records, such as age, height, weight, total bilirubin (TBIL), direct bilirubin (DBIL), indirect bilirubin (IBIL), baseline alanine aminotransferase (ALT), baseline aspartate transaminase (AST), sex, UGT1A1*28 mutation type and UGT1A1*6 mutation type. Other covariates were those from further calculated, including BMI, body surface area (BSA), lean body weight (LBW), body fat rate (BF%), endogenous creatinine clearance rate (CLcr), ideal body weight (IBW), adjusted weight and adjusted CLcr. BMI was calculated by the World Health Organization (WHO) admitted formula (Keys et al., 1972), and the BSA was obtained by applying Du Bios’ formula (Du Bois and Du Bois, 1916). The LBW and BF% were considered as other covariates and added to the PopPK model because of the high lipid solubility of the drugs (Park et al., 2018). The Cockcroft-Gault formula (Eq. 4) was used to figure out the CLcr (Cockcroft and Gault, 1976). Additional covariates such as IBW (Eq. 5) (McCarron et al., 1974; Robinson et al., 1983), adjusted weight (Eq. 6) and adjusted CLcr (calculated by adjusted weight for BMI >25 kg/m^2^) were introduced into the model (Winter et al., 2012).

For males:

$$CLcr\left(mg/dL\right)=\frac{\left(140-age\left(years\right)\right)*weight\left(kg\right)*88.4}{72*serum\;creatinine\left(\mu mol/L\right)}$$ (4)

$$IBW\left(kg\right)=50+2.3*\left(\frac{height\left(cm\right)}{2.54}-60\right)$$ (5)

$$adjusted\;weight\left(kg\right)=\left(weight\left(kg\right)-IBW\left(kg\right)\right)*0.4+IBW\left(kg\right)$$ (6)

For females:

$$CLcr\left(mg/dL\right)=\frac{\left(140-age\left(years\right)\right)*weight\left(kg\right)*88.4}{72*serum\;creatinine\left(\mu mol/L\right)}\;*0.85$$ (4a)

$$IBW\left(kg\right)=48.67+1.65*\left(\frac{height\left(cm\right)}{2.54}-60\right)$$ (5a)

$$adjusted\;weight\left(kg\right)=\left(weight\left(kg\right)-IBW\left(kg\right)\right)*0.4+IBW\left(kg\right)$$ (6a)

The effect of continuous covariates was described by the power function after the normalization using the population median (Eq. 7), and the effect of categorical covariates was modeled using the exponential function (Eq. 8 **)**:

$${Effect}_{i}={\left(\frac{{Cov}_{ij}}{{Cov}_{median}}\right)}^{\theta_{covi}}$$ (7)

$${Effect}_{i}=e^{{Cov}_{ij}{*\theta }_{covi}}$$ (8)

where ${Effect}_{i}$ is the multiplicative factor of the covariate i, ${Cov}_{ij}$ is the continuous covariate value or categorical variable with the value of 0 or 1 for the covariate i in individual j, ${Cov}_{median}$ is the median value of covariate, and $\theta_{covi}$ describe the fixed effect for covariate i.

However, not all the covariates mentioned above were applied to construct the final covariate model in order to avoid the presence of covariate collinearity. In the univariate screening process, when both two covariates had a significant impact on the same pharmacokinetic parameter and they were highly correlated (r > .5), such as CLcr and adjusted CLcr, only one of them was reserved in the model. The covariates that remained were selected or excluded utilizing forward and backward stepwise on the basis of the change of OFV value. If the decrease of OFV exceeded 3.84 points (*p* < .05, df = 1), the covariates could join the basic model in the forward inclusion process. Subsequently, backward elimination was employed to confirm the covariate selection. If the increase of OFV was less than 6.64 points (*p* < .01, df = 1), the covariates should be retained in the final PopPK model. Moreover, the correlation between IIV of pharmacokinetic parameters should be clarified to construct a covariance model.

### 2.6 Model validation

Goodness-of-fit (GOF) plots, visual predictive check (VPC), non-parametric bootstrap and test of normalized distribution errors (NPDE) were adopted to confirm the validity of the final model. The GOF plots evaluated the reliability of the final model, including drug concentration observations (DV) *versus* population predictions (PRED) or individual predictions (IPRED) and conditional weighted residuals (CWRES) *versus* time or PRED. VPC was performed by simulating 1,000 virtual data per time in the final model based on Monte Carlo simulation and the model predictions and DV at the 5th, 50th and 95th percentiles were compared. The bootstrap analysis was implemented to judge the robustness of the last model. During this analysis, the initial dataset was randomly resampled 1,000 times with replacement, and then the obtained 95% confidence intervals of pharmacokinetic parameters were compared with the typical value of parameters in the final model. NPDE values, which were supposed to obey standard normal distribution if the final model was effective, were acquired after the 1,000 times simulation of each subject observation.

### 2.7 Model application

This final PopPK model was used to predict TQ-B3203 exposure (AUC_0-t_ and C_max_), with the pharmacokinetic parameters counted by individual Bayes estimates. The sensitive plots were painted to clarify the effect of an identified covariate on the exposure parameters. Considering the occurrence of adverse effects, the single dose at 25 mg/m^2^ level was finally chosen as a simulated dosing regimen in a representative population (with the median values of continuous covariates considered as the typical value). When the impact of one identified covariate was evaluated, the value of this covariate was regarded as the 5th or 95th percentiles of the population, with other covariates fixed to the typical value. The general exposure under the influence of covariates was compared with the exposure of the typical population.

## 3 Results

### 3.1 Clinical data summary

A total of 316 TQ-B3203 concentrations from 15 subjects with 25 episodes were used in the PopPK modeling. The demographic and clinical characteristics of baseline continuous and categorical covariates gathered from subjects are summarized in Table 1.

**TABLE 1:** Baseline demographic and clinical characteristics^a^.

Characteristic	Median (IQR)	Range
No. of patients	15	
Sex, male/female	10 (66.7%)/5 (33.3%)	
UGT1A1*28, TA (6)/TA (6); TA (6)/TA (7)	14 (93.3%)/1 (6.7%)	
UGT1A1*6, 211G/G; 211G/A	10 (66.7%)/5 (33.3%)	
		
Treatment episode	25	
No. of concentration	316	
Patient age, years (n = 25)	57 (44–65)	31–70
Height, cm (n = 25)	164 (160–173)	148–178
Weight, kg (n = 25)	64.0 (57.5–68.0)	47.9–80.0
Adj weight, kg (n = 25)	61.90 (55.55–70.57)	48.21–74.31
IBW, kg (n = 25)	60.50 (54.26–68.65)	45.81–73.18
LBW, kg (n = 25)	49.53 (38.36–55.45)	32.09–59.40
BF% (n = 25)	23.39 (20.94–33.01)	13.41–41.51
BMI, kg/m^2 (n = 25)	23.44 (21.05–24.91)	18.64–28.97
BSA, m^2 (n = 25)	1.678 (1.600–1.841)	1.420–1.938
CRE, μmol/L (n = 25)	60 (51–69)	38–204
CLcr, mg/dl (n = 25)	109.2 (83.64–120.0)	27.35–157.3
Adj CLcr, mg/dl (n = 25)	101.9 (83.64–113.9)	27.35–157.3
TBIL, μmol/L (n = 25)	9 (5.4–10.6)	2.9–25.8
DBIL, μmol/L (n = 25)	2.82 (2.3–4)	1.5–7.9
IBIL, μmol/L (n = 25)	6 (2.9–7.78)	.9–17.9
ALT, IU/L (n = 25)	11.3 (8–17)	3–24
AST, IU/L (n = 25)	16.7 (13.2–21)	11.2–29

^a^ IQR, interquartile range; No. of patients, numbers of patients; No. of concentration, numbers of concentration; Adj weight, adjusted weight; IBW, ideal body weight; LBW, lean body weight; BF%, body fat percentage; BMI, body mass index; BSA, body surface area; CRE, serum creatinine; CLcr, endogenous creatinine clearance rate; Adj CLcr, adjusted endogenous creatinine clearance rate; TBIL, total bilirubin; DBIL, direct bilirubin; IBIL, indirect bilirubin; ALT, baseline alanine aminotransferase; AST, baseline aspartate transaminase.

### 3.2 Pharmacokinetic analysis

**FIGURE 1:**
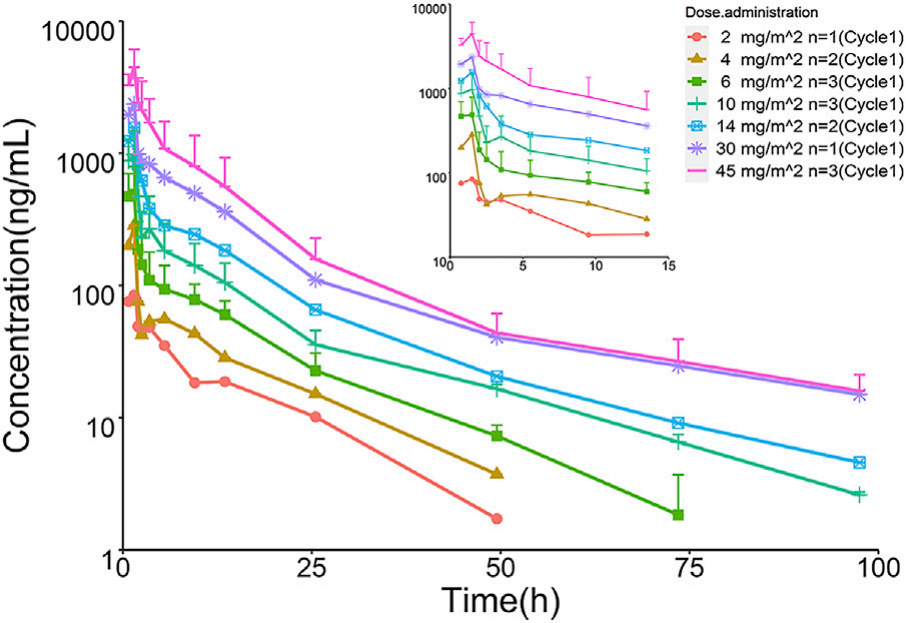
Semilogarithmic plots of mean concentration *versus* time of TQ-B3203.

**FIGURE 2:**
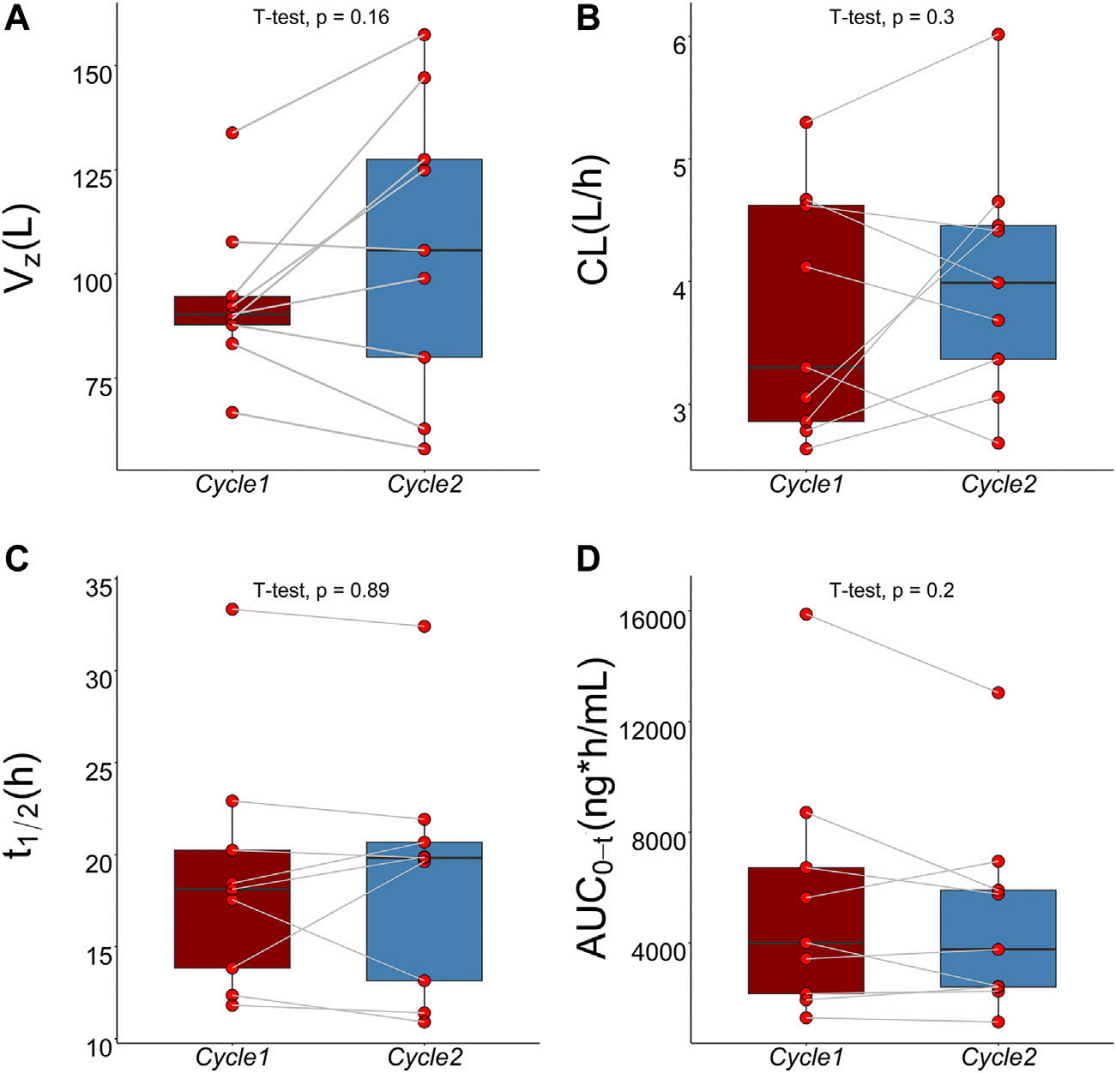
Comparison of pharmacokinetic parameters between cycle 1 and cycle 2 using paired *t*-test. **(A)** Comparison of V_z_ between cycle 1 and cycle 2, **(B)** Comparison of CL between cycle 1 and cycle 2, **(C)** Comparison of t_1/2_ between cycle 1 and cycle 2, **(D)** Comparison of AUC_0-t_ between cycle 1 and cycle 2.

The mean plasma TQ-B3203 concentration *versus* time semilogarithmic plot at different levels is displayed in Figure 1. The corresponding pharmacokinetic parameters are presented in Table 2. C_max_ always appears at the end of intravenous injection (1.5 h), followed by an obvious rapid elimination phase and a slow elimination phase. The half-life of TQ-B3203 is about 10–41 h (n = 15, median = 19.62, mean = 20). There is no significant difference in pharmacokinetic parameters between cycles 1 and 2 shown in Figure 2. Furthermore, an obvious accumulation of TQ-B3203 was not found in these dose groups.

**TABLE 2:** Main pharmacokinetic parameters of TQ-B3203 calculated by NCA.

	Dose	AUC_0-t_ ^a^ (ng·h/mL)	AUC_0-inf_ ^a^ (ng·h/mL)	C_max_ ^a^ (ng/mL)	T_max_ ^b^ (h)	t_1/2_ ^a^ (h)	V_z_ ^a^ (L)	CL^a^ (L/h)
**cycle 1**	2 (n = 1)	775	801	85	1.5	10.3	68.1	4.6
	4 (n = 2)	1,410	1,423	284	1.5 (1.5.1.5)	11.3	77.3	4.7
	6 (n = 3)	2705 (1,133)	2778 (1,121)	533 (236)	1.5 (.75.1.5)	16.4 (5.7)	90 (6.0)	4.0 (1.0)
	10 (n = 3)	5258 (1,679)	5326 (1,676)	1,044 (507)	1.5 (.75.1.5)	18.0 (.5)	87.4 (20.5)	3.4 (.7)
	14 (n = 2)	8584	8731	1,559	1.5 (1.5.1.5)	21.6	90	2.9
	30 (n = 1)	15882	16602	2357	1.5	33.3	133.8	2.8
	45 (n = 3)	26251 (11761)	27026 (11688)	4956 (787)	1.5 (.75.1.5)	34.3 (5.9)	148.7 (86.1)	2.9 (1.2)
**cycle 2**	4 (n = 2)	1,142	1,154	229	1.5	11.4	99.0	6.0
	6 (n = 3)	2366 (98)	2399 (102)	412 (126)	1.5 (.75.1.5)	17.5 (5.8)	111.6 (44)	4.4 (.3)
	10 (n = 3)	5493 (1,611)	5571 (1,608)	1,187 (687)	1.5 (.75.1.5)	17.9 (4.1)	81.2 (23.9)	3.1 (.5)
	14 (n = 1)	5901	5954	1,140	1.5	19.8	127.5	4.5
	30 (n = 1)	13040	13633	1,680	1.5	32.4	157.4	3.4

^a^ The data are shown as mean (SD).

^b^ T_max_ is shown as median (minimum, maximum).

### 3.3 PopPK model development

**FIGURE 3:**
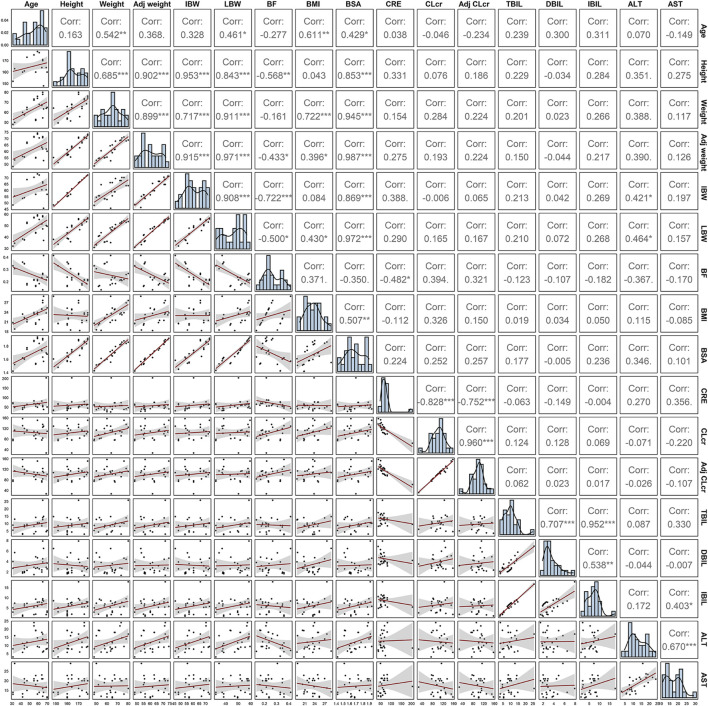
Spearman correlation analysis of continuous covariates.

**FIGURE 4:**
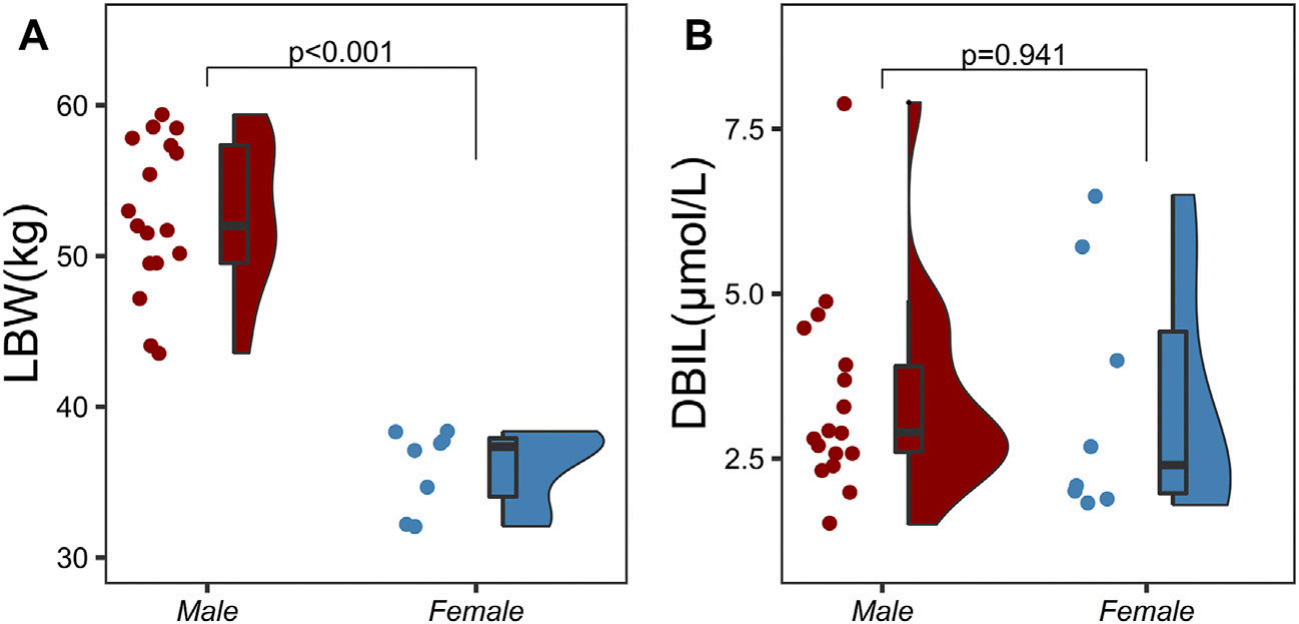
Correlation analysis between categorical variables and continuous variables using two independent samples *t*-test. **(A)** Correlation analysis between sex and lean body weight (LBW), **(B)** Correlation analysis between sex and direct bilirubin (DBIL).

A three-compartment model with first-order elimination, which was parameterized as the V_1_, the shallow peripheral volume of distribution (V_2_), the deep peripheral volume of distribution (V_3_), the CL and the inter-compartment clearance (CL_2_ and CL_3_), was selected as the best structural model to describe the pharmacokinetic profiles of TQ-B3203. The optimal model evaluation results were acquired in the selection of an exponential model to characterize the inter-individual variability and a log-additive model to describe intra-individual variability, so the pharmacokinetic parameters were estimated with the natural logarithm-transformed (log) plasma TQ-B3203 concentration data. IOV was not included in the model, because after the incorporation of IOV on CL and V_1_, the log-additive error changed little from 22.4% to 21.4% and the fitting degree of the subsequent model was always poor in models, although there was a statistically significant decrease in OFV. Actually, there was no significant difference in pharmacokinetic parameters between cycles 1 and 2, so it could be considered that the cycle did not significantly affect the pharmacokinetic behavior of the TQ-B3203 *in vivo*. The distribution and correlation of continuous covariates are shown in Figure 3. The categorical covariate sex and continuous covariates such as LBW and DBIL were considered to have significant influences on the same pharmacokinetic parameters simultaneously, and the correlation between them required to be examined (shown in Figure 4). Two covariates with a correlation coefficient greater than .5 were refrained from containing in the covariate screening process simultaneously in order to avoid the covariate collinearity. So only adjusted CLcr, BMI, UGT1A1*28 genotype, UGT1A1*6 genotype, DBIL, ALT and LBW, both uncorrelated to each other, were utilized for covariate screening after the collinearity check. The results of forward and backward stepwise procedures are presented in detail in Table 3. The OFV significant decreases of adding BMI, DBIL and LBW to covariate models indicated that both three covariates had significant impacts on the pharmacokinetic parameters of TQ-B3203. The computational formulas of pharmacokinetic parameters V_1_ and CL in the final model are shown as follows:

$$\begin{array}{l} V_{1}\left(L\right)=4.81*{\left(\frac{LBW}{49.53}\right)}^{1.18}*e^{\eta V1}\\ \; \end{array}$$ (9)

$$\begin{array}{c} CL\;(L/h)=3.97*{\left(\frac{BMI}{23.44}\right)}^{0.78}*{\left(\frac{DBIL}{2.82}\right)}^{-0.24}*e^{\eta CL} \end{array}$$ (10)

where 4.81 L and 3.97 L/h are typical values of V_1_ and CL, respectively. The median values of significant covariates LBW, BMI and DBIL are 49.53 kg, 23.44 kg/m^2^ and 2.82 μmol/L. The estimated correlation coefficients such as 1.18, .78 and −.24, represent the relationship between LBW and V_1_, BMI and CL, DBIL and CL, respectively. The final PopPK parameters estimations are summarized in Table 4, which were obtained with satisfactory precision (RSE%<38%) and within the 95% confidence interval of bootstrap results.

**TABLE 3:** Results of the forward and backward stepwise procedure^a^.

Step	Covariate screening	OFV	ΔOFV	p-value	Comments
1	None	160.054			Base model
Forward inclusion					
2	V_2_-LBW	145.503	−14.551	<.001	
3	CL_2_-DBIL/V_2_-LBW	135.132	−10.371	<.01	
4	CL_2_-DBIL-LBW/V_2_-LBW	123.487	−11.645	<.001	
5	CL_2_-DBIL-LBW/V_2_-LBW/V_1_-LBW	118.287	−5.2	<.05	
6	CL_2_-DBIL-LBW/V_2_-UGT1A1*6-LBW/V_1_-LBW	111.601	−6.686	<.01	
7	CL-BMI/CL_2_-DBIL-LBW/V_2_- UGT1A1*6-LBW/V_1_-LBW	104.794	−6.807	<.01	
8	CL-BMI-DBIL/CL_2_-DBIL-LBW/V_2_- UGT1A1*6-LBW/V_1_-LBW	93.483	−11.311	<.001	Full model
Backward elimination					
9	CL-BMI-DBIL/CL_2_-DBIL-LBW/V_2_-LBW/V_1_-LBW	97.753	4.27	>.01	Final model

^a^ ΔOFV, the change of OFV.

**TABLE 4:** Pharmacokinetics parameter estimate in the final model and bootstrap results^a^.

Parameters	Final model		Bootstrap	
	Estimate (%RSE)	95% CI	Median (%RSE)	95% CI
V_1_ (L)	4.81 (8.47%)	4.01–5.61	4.83 (9.05%)	4.05–5.87
V_2_ (L)	24.44 (5.94%)	21.58–27.30	24.69 (8.12%)	21.49–29.37
V_3_ (L)	27.98 (6.69%)	24.30–31.66	27.81 (7.79%)	24.07–32.29
CL (L/h)	3.97 (4.85%)	3.59–4.35	3.96 (5.01%)	3.58–4.33
CL_2_ (L/h)	1.95 (20.48%)	1.17–2.74	1.96 (23.87%)	1.28–3.13
CL_3_ (L/h)	10.58 (11.28%)	8.23–12.92	10.45 (11.42%)	8.32–13.06
BMI on CL (L/h)	.78 (22.08%)	.44–1.12	.79 (39.13%)	.05–1.25
DBIL on CL (L/h)	−.24 (−36.01%)	−.42 to −.07	−.26 (−36.45%)	−.44 to −.09
DBIL on CL_2_ (L/h)	−1.77 (−19.55%)	−2.46 to −1.09	−1.81 (−23.12%)	−2.68 to −1.04
LBW on CL_2_ (L/h)	−2.55 (−27.03%)	−3.90 to −1.19	−2.53 (−31.22%)	−4.18 to −1.14
LBW on V_1_ (L)	1.18 (37.01%)	.32–2.05	1.22 (44.96%)	.19–2.36
LBW on V_2_ (L)	−1.41 (−19.49%)	−1.95 to −.87	−1.39 (−28.02%)	−1.92 to −.41
Inter-individual variability				
ω^2^CL	.043 (36.00%)	.013–.073	.039 (39.06%)	.012–.071
ω^2^V_3_	.117 (29.88%)	.048–.185	.103 (35.64%)	.039–.185
ω^2^CL_2_	.573 (32.60%)	.203–.944	.529 (35.43%)	.173–.918
ω^2^CL_3_	.290 (32.50%)	.103–.477	.251 (34.40%)	.092–.424
Residual variability (σ)				
stdev0	.200 (10.30%)	.159–.240	.198 (10.43%)	.160–.239

^a^ RSE, relative standard error; CI, confidence interval; ωCL, variance of inter-individual variability for CL; ωV_3_, variance of inter-individual variability for V_3_; ωCL_2_, variance of inter-individual variability for CL_2_; ωCL_3_, variance of inter-individual variability for CL_3_; stdev0, standard deviation.

### 3.4 Model validation

**FIGURE 5:**
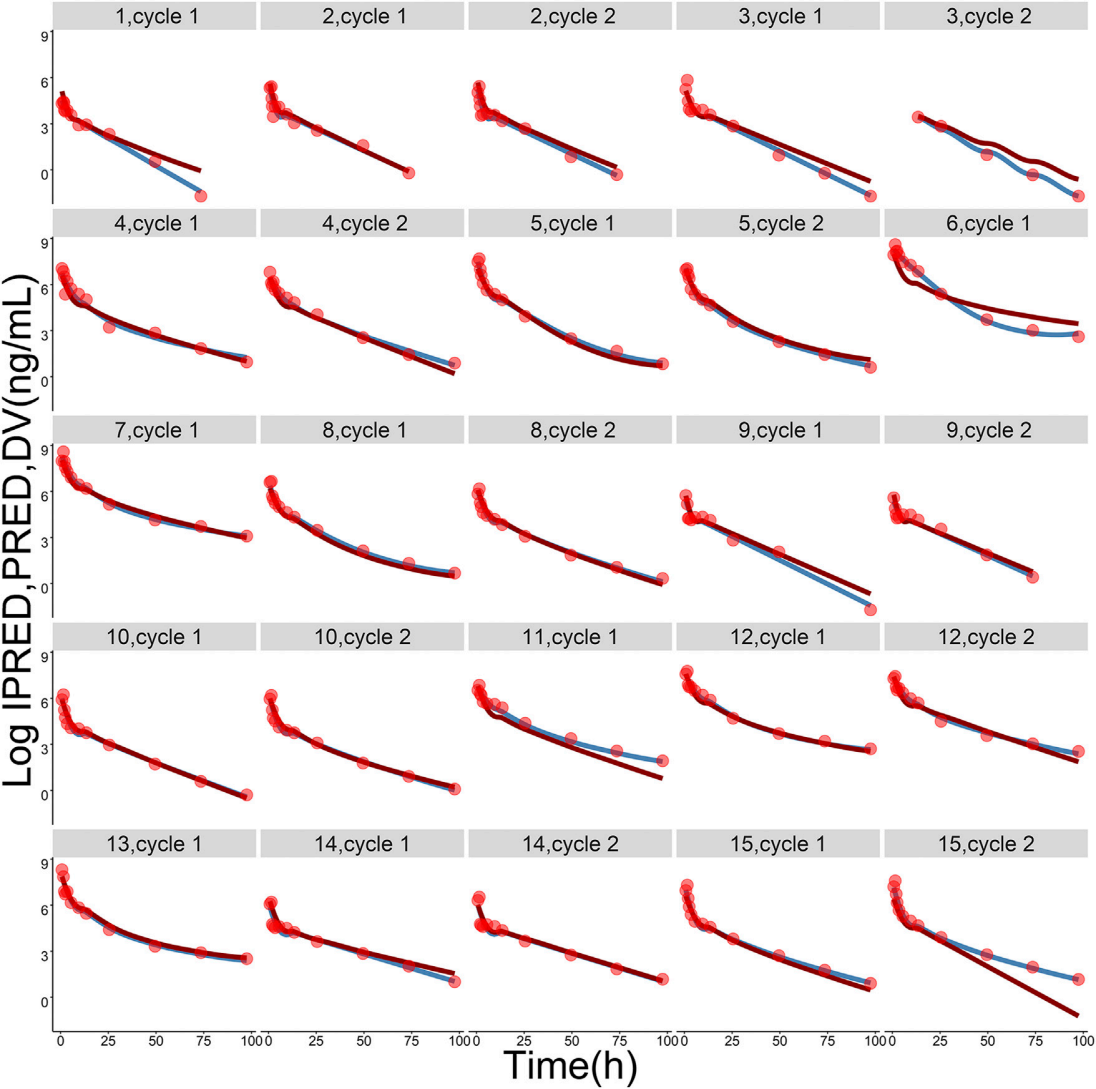
The logarithmic-transformed observations (log DV, red dots), logarithmic-transformed population predictions (log PRED, dark-red lines) and logarithmic-transformed individual predictions (log IPRED, steel-blue lines) from the final population pharmacokinetic model. The gray label on the top of each picture represents ID of each subject and treatment cycle.

**FIGURE 6:**
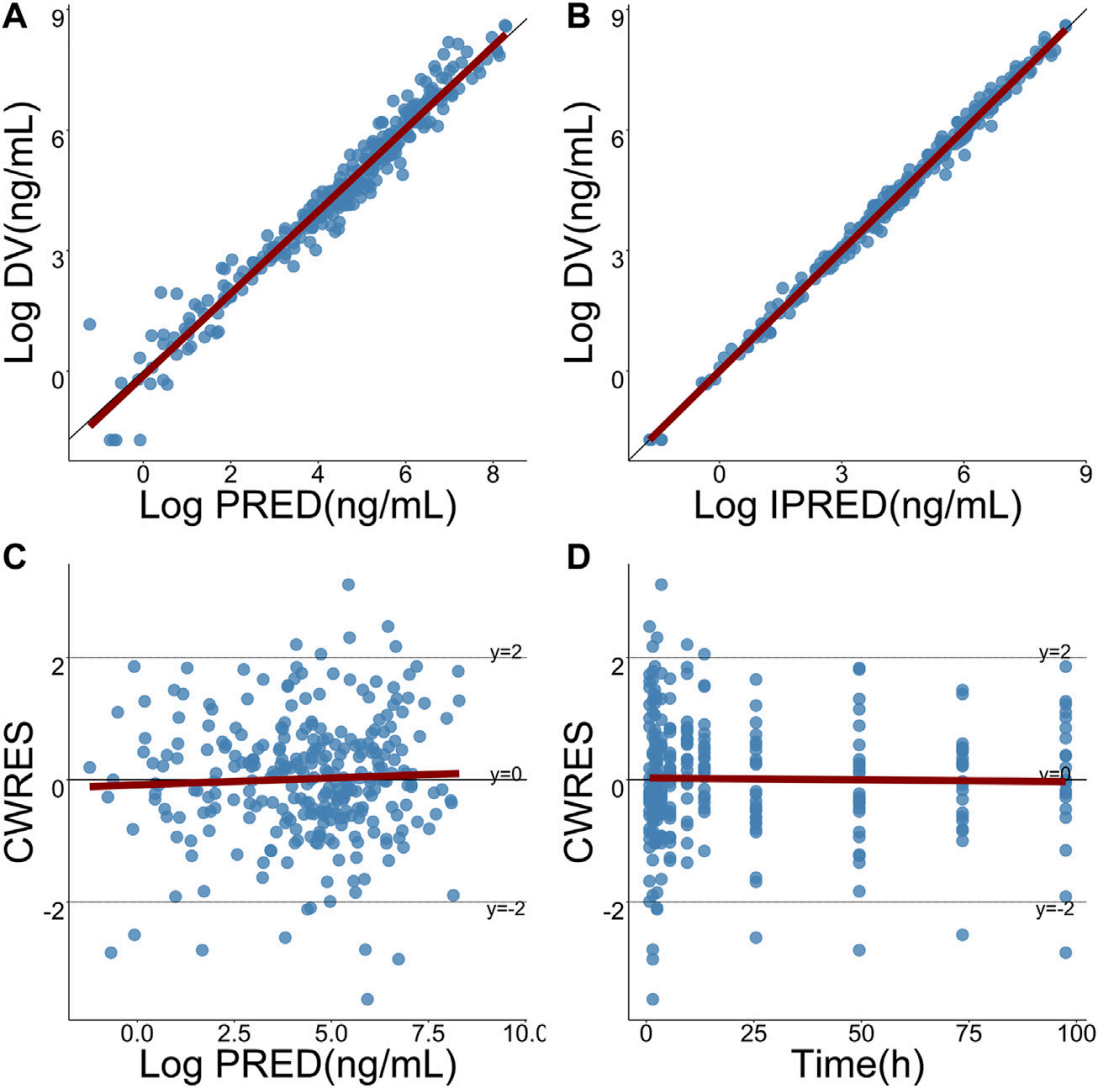
Goodness-of-fits plots for the final model. **(A)** Logarithmic-transformed observations (log DV) of TQ-B3203 *versus* logarithmic-transformed population predictions (log PRED), **(B)** Log DV *versus* logarithmic-transformed individual predictions (log IPRED), **(C)** Conditional weighted residual (CWRES) *versus* log PRED, **(D)** CWRES *versus* time. The dark-red lines represent linear regression.

**FIGURE 7:**
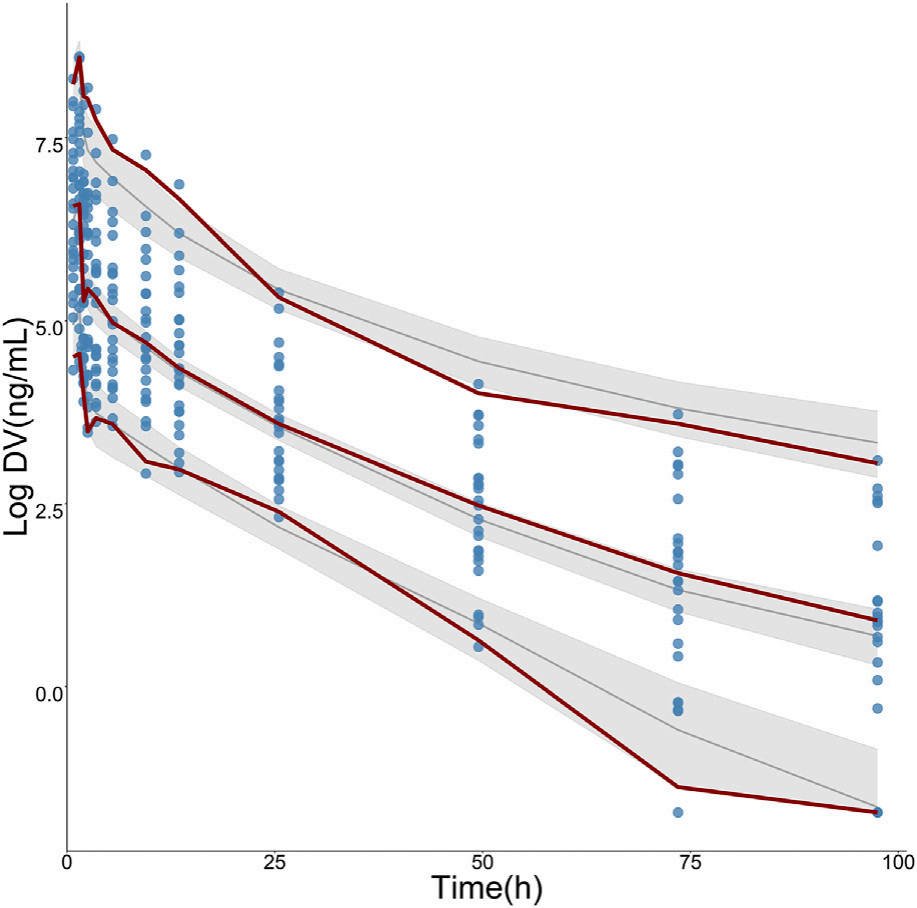
Visual predictive check (VPC) for logarithmic-transformed observations (log DV) of TQ-B3203. The steel-blue points represent log DV, while the dark-red dotted lines represent the 5^th^, 50th, and 95th percentiles of log DV. The solid black lines show the 5^th^, 50th, and 95th percentiles of simulated results, and the shaded regions represent the 95% confidence intervals for medians (solid black lines), respectively.

**FIGURE 8:**
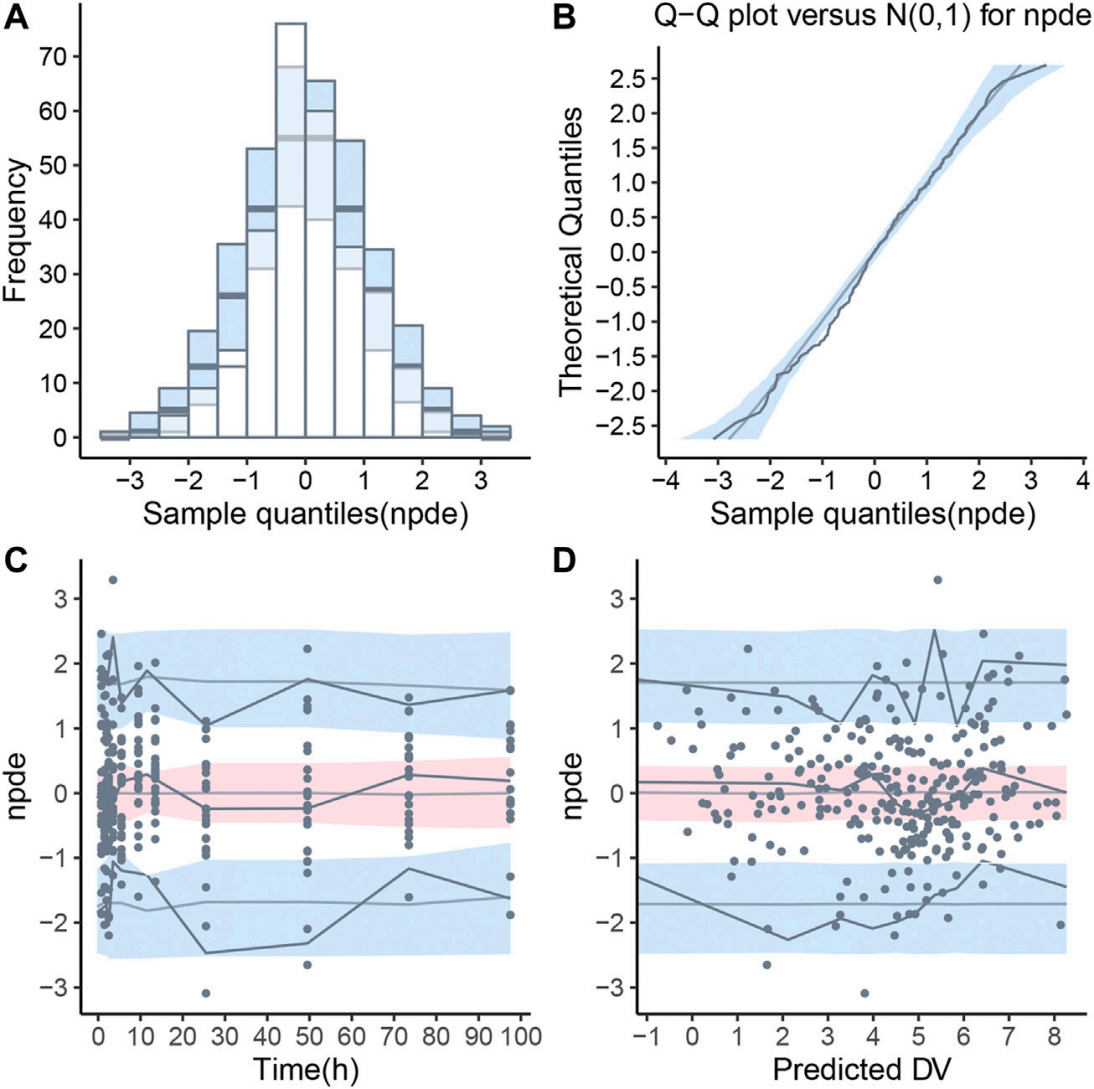
Normalized prediction distribution error (NPDE) for final population pharmacokinetic model. **(A)** Histogram of NPDE distribution with the density of the theoretical standard normal distribution (semi-transparent blue fields), **(B)** Quantile-quantile plot of NPDE against expected standard normal distribution (semi-transparent blue fields), **(C)** Scatterplot of NPDE *versus* time, **(D)** Scatterplot of NPDE *versus* population predictions (PRED). These two scatterplots showed the observations as blue dots, the median observations as solid red lines, the 5th and 95th percentiles of observations as solid blue lines, and 95% confidence intervals of relevant forecast percentiles as the red or blue fields.

Pharmacokinetic curves of treatment episodes were predicted using the final model. The result, shown in Figure 5, indicated that the log IPRED profiles could almost entirely describe the log DV. The final model was evaluated by diagnostic plots shown in Figure 6, which suggested that the model was of good fit. The log DV *versus* log PRED or log IPRED was close-to-symmetrically distributed by the reference y = x, and the majority of CWRES *versus* log PRED or time were randomly distributed between −2 and +2 with no obvious bias. The VPC plots in Figure 7 indicated that the final model had excellent prediction performance because the vast majority of log DV were contained within the model-based simulated confidence intervals. All pharmacokinetic parameters estimations of the final model were included in the 95% confidence interval computed from the non-parametric bootstrap, listed in Table 4. As Figure 8 showed, the NPDE was considered a normal distribution and variance homogeneity, and the NPDE *versus* time or prediction had no apparent tendency to deviate from specified intervals. Furthermore, a statistical summary of the NPDE value distribution demonstrated that the mean did not significantly differ from 0 (Student’s t-test, *p* = .591), variance had no remarkable difference from 1 (Fisher test, *p* = .265), and NPDE distribution was considered to be a standard normal distribution (Shapiro-Wilks test of normality, *p* = .539) so that the final model was appropriate to describe these observations. The results above indicated that the final model achieved the right qualifications for predicting and assessing pharmacokinetics in this group of patients.

### 3.5 Model application

**FIGURE 9:**
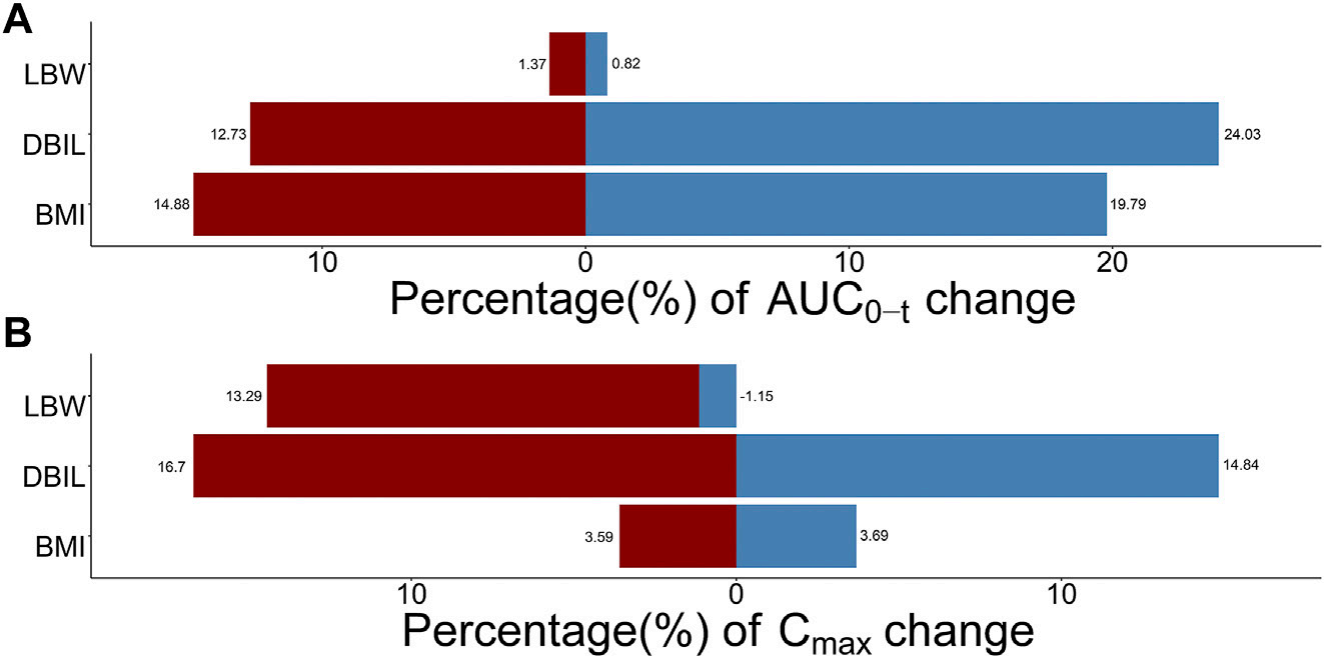
Sensitive analysis plots comparing the influence of covariates on TQ-B3203 exposure (AUC_0-t_ and C_max_). **(A)** The effect of covariates on AUC_0-t_, **(B)** The effect of covariates on C_max_. The percentage of change is calculated compared with a simulated typical population whose AUC_0-t_ is 9909.74 ng*h/mL and C_max_ is 1824.91 ng/mL.

The influence of significant covariates on TQ-B3203 predicted exposure (AUC_0-t_ and C_max_) is presented in Figure 9. The percentage of AUC_0-t_ and C_max_ change was calculated compared with a simulated typical subject whose AUC_0-t_ and C_max_ were 9909.74 ng*h/mL and 1824.91 ng/mL, respectively. These 2 bar charts indicated that LBW had a great impact (14.44%) on C_max_ but little (2.19%) on AUC_0-t_. On the contrary, BMI exerted a considerable influence (34.67%) on AUC_0-t_ but a small (7.28%) on C_max_. The significance of DBIL on both AUC_0-t_ (36.76%) and C_max_ (31.54%) was similarly excellent.

## 4 Discussion

This research aimed to establish a PopPK model to find the potential covariates of inter-individual variation because highly variable pharmacokinetic characteristics of TQ-B3203 were observed in the phase I clinical trial. According to the results, a three-compartment model with first-order elimination was considered as the optimal structural model to characterize concentration data, in which the typical CL and V_1_ were estimated as 3.97 L/h and 4.81 L, respectively. DBIL and BMI were the two most influential factors on CL, and LBW was considered to affect V_1_ following the stepwise covariate modeling process. Furthermore, LBW was found to be related to V_2_ as well as CL_2_, and CL_2_ was also influenced by DBIL.

In the previous PopPK analysis of irinotecan, the SN38 (a metabolite of irinotecan) concentration was also included to build a combined model, because irinotecan is a prodrug and SN38 showed a 300–1,000 times higher activity than irinotecan (Berg et al., 2015; Adiwijaya et al., 2017; Oyaga-Iriarte et al., 2019; Brendel et al., 2021; Liu et al., 2022). In this study, although TQ-B3203 also could be metabolized to produce SN38, the conversion ratio was very low (<5%), and TQ-B3203 mainly existed in the form of the prototype *in vivo*. It is reasonable that only the TQ-B3203 concentration was used in the modeling procedure.

BMI is the most extensively used indicator of judging whether an individual is thin, overweight or obese. Generally, the bigger the BMI value is, the fatter the person is, and the increasing BMI value or obesity will cause slow distribution, prolonged half-life and relatively decreased CL of lipophilic drugs due to its high tendency to distribute in adipose tissue (Powis et al., 1987). However, obesity could also boost phase I and phase II metabolism procedures, thus resulting in increased drug CL (Abernethy et al., 1983; Morgan and Bray, 1994; Marik and Varon, 1998; Brill et al., 2012). In this study, BMI was positively correlated with CL, which was consistent with other fat-soluble drugs, such as tigecycline and dilmapimod (Xie et al., 2017; Yang and Dumitrescu, 2017).

LBW could quantify the variation of renal and hepatic CL for individuals and provide the basis for the conceptual transformation of the relationship between body components and CL (Han et al., 2007). Although the relationship between LBW and CL was theoretically strong, it was not found in this study, which may be due to insufficient samples (Morgan and Bray, 1994). In the present model, LBW was the only significant covariate for V_1_ and larger LBW was associated with greater V_1_.

In the preclinical study, TQ-B3203 was found to be excreted into feces through bile in the form of the prototype. The impaired bile excretion function will lead to the increase of DBIL level and the decrease of TQ-B3203 CL, so DBIL was negatively correlated with TQ-B3203 CL, which could be identified in this study. In addition, other liver function-related indicators such as TBIL, IBIL, ALT and AST were all considered to be included in this covariate analysis at first. After the univariate screening process, only DBIL and ALT were retained in the final covariate selection to avoid covariate collinearity, but we did not find evidence of ALT as a significant covariate on CL.

As a study to establish a PopPK model of a novel antitumor agent using the data obtained from early clinical trials, there is a defect that is not neglected. Due to the limited number of subjects in the dose-escalating stage of the phase I clinical trial, the range of covariates provided by the population was narrow, so the extrapolation of research results was restricted. However, the intensive sampling points of every subject could offer detailed preliminary data on the pharmacokinetics of advanced solid tumor patients. This could make up for the defect of a small number of subjects to some extent and improve the significance of our model. This study is the first PopPK analysis of TQ-B3203, more extensive research is needed to take place to identify clinically relevant covariates of TQ-B3203 pharmacokinetics. The significant covariates obtained in this study, such as LBW, BMI and DBIL, suggested that weight and liver function related covariates may be the important factors affecting the pharmacokinetics of TQ-B3203, which could provide references for subsequent studies.

## 5 Conclusion

The first robust PopPK model of TQ-B3203 was successfully generated following intravenous administration of TLI in Chinese patients with advanced solid tumors. BMI, LBW, and DBIL were significant covariates that affected the pharmacokinetics of TQ-B3203. The final model was applied to predict the influence of significant covariates on drug exposure. In a word, this PopPK model could provide the references for the dose regimen in the future study of TLI.

## Data Availability

The original contributions presented in the study are included in the article/Supplementary Materials, further inquiries can be directed to the corresponding author.
