# Supplementary material for: Population pharmacokinetic analysis of TQ-B3203 following intravenous administration of TQ-B3203 liposome injection in Chinese patients with advanced solid tumors
Source: Front Pharmacol. 2023 Jan 16;14:1102244. doi: 10.3389/fphar.2023.1102244 (PMC9885713; doi:10.3389/fphar.2023.1102244)
Supplement: Supplementary file 1 [file DataSheet1.DOCX]

***Supplementary Material***


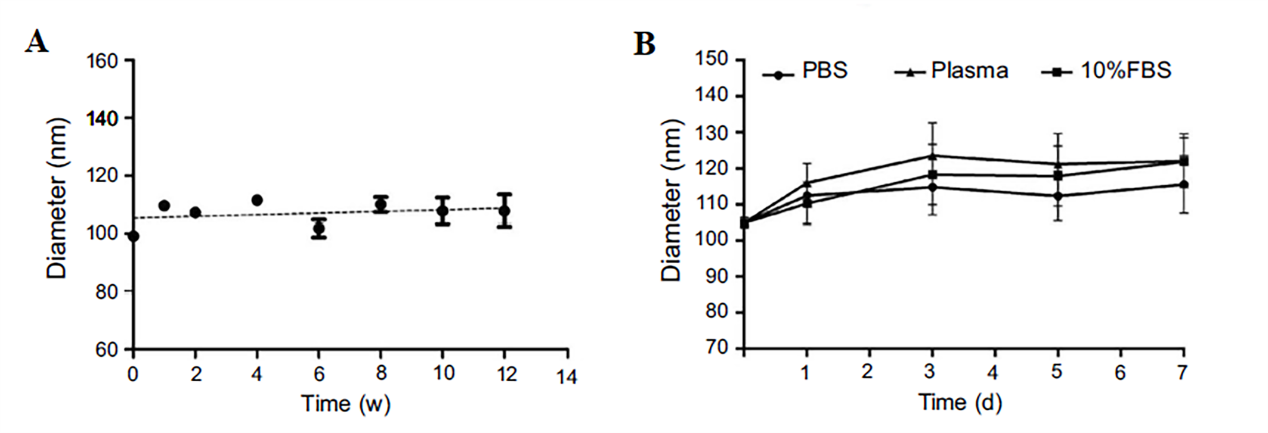


**Figure S1** The formulation-related stability of TQ-B3203. (A) Size changes of TQ-B3203 liposome were examined as a function of time at 4°C for 3 months, (B) Size changes of TQ-B3203 liposome in phosphate buffered saline (PBS), plasma and fetal bovine serum (FBS) were examined for 7 days.


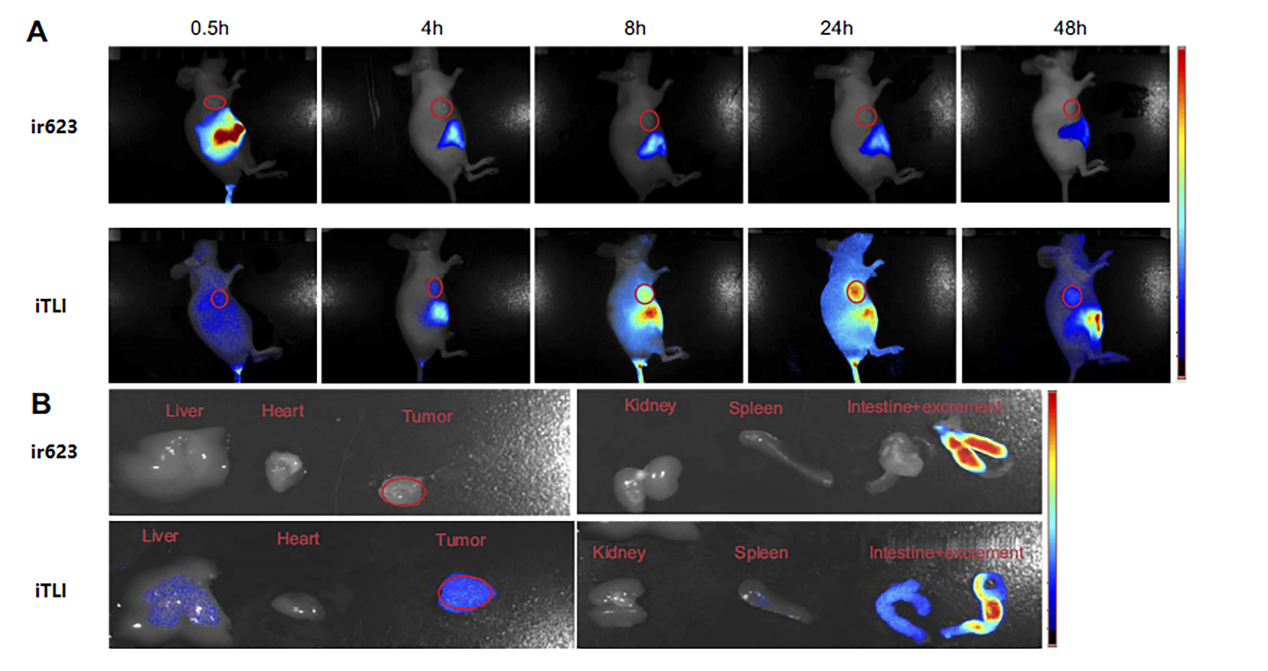


**Figure S2** Biodistribution of ir623 and near-infrared region probe-embedded TQ-B3203 liposome injection (iTLI) in HT-29 tumor-bearing mice. (A) Representative images of in vivo whole-body imaging of mice at 0.5, 4, 8, 24, and 48 hours post-intravenous injection of ir623 and iTLI, (B) The optical images of the tumors and other major organs from the sacrificed mice (n=3).





**Figure S3** The structures of TQ-B3203 (A) and internal standard TQ-B3203-d_8_ (B).
